# Incidental finding of large pneumothorax on Cardiac MR scan

**DOI:** 10.1186/s12880-017-0240-6

**Published:** 2018-02-12

**Authors:** J. P. M. Andrews, G. McKillop, M. R. Dweck

**Affiliations:** 1 Centre for Cardiovascular Sciences, Chancellors Building, University of Edinburgh, 49 Little France Crescent, Edinburgh, EH16 4SB UK; 2 Edinburgh Heart Centre, Royal infirmary of Edinburgh, 49 Little France Crescent, Edinburgh, EH16 4SA UK; 3 Department of Radiology, Royal Infirmary of Edinburgh, 49 Little France Crescent, Edinburgh, EH16 4SA UK

**Keywords:** Cardiac magnetic resonance imaging, Pneumothorax, CMR, Thoracic MR, Incidental findings

## Abstract

**Background:**

We believe this is the first case report of a pneumothorax being identified using cardiac magnetic resonance imaging. This case also illustrates the haemodynamic effect a large pneumothorax can have on right ventricular filling in diastole.

**Case presentation:**

A 26-year-old attended for an interval follow up Cardiac Magnetic Resonance (CMR) of his thoracic aorta after a thoracic co-arctation repair aged 3. He was found to have an incidental large pneumothorax by the reporting cardiology fellow which was confirmed by the on-call radiologist. The pneumothorax was most notable for its compression of the right ventricle in diastole. Although the patient had worrying features on CMR imaging, he remained clinically stable and a conservative approach to management saw the pneumothorax resolve after a 3 week period.

**Conclusions:**

Pneumothoraces are important, potentially life threatening conditions. Although very rarely identified on MR imaging, radiographers and reporting doctors should be aware of their key features. This case serves to identify not only the abnormal lung parenchymal features but also the striking compressional effect of the pneumothorax on the right ventricle in diastole. Indeed we believe this is the first case report of a pneumothorax identified on CMR imaging.

**Electronic supplementary material:**

The online version of this article (10.1186/s12880-017-0240-6) contains supplementary material, which is available to authorized users.

## Background

Pneumathoraces are potentially life threatening causes of chest pain with a rapid diagnosis critical to improving the chances of a positive outcome. They classically present with acute pleuritic chest pain and dyspnoea with the diagnosis confirmed on chest X ray. Treatment may be conservative or involve needle decompression depending on individual clinical impact. Although very rarely identified on MR imaging, supervising staff and reporting doctors should be aware of their key features. This case serves to identify not only the abnormal lung parenchymal features but also the striking compressional effect of a pneumothorax on the right ventricle during both systole and diastole. Indeed, we believe these are the first published images of a pneumothorax identified on CMR.

## Case presentation

A 26-year-old presented for follow up Cardiac Magnetic Resonance (CMR) of his thoracic aorta. He had undergone an end-to-end anastomosis repair of a thoracic aortic co-arctation aged 3. His last CMR scan 7 years previously had demonstrated normal appearances to the repair. The patient had not reported any symptoms at a recent clinic appointment and did not complain of any symptoms when he attended for imaging. He underwent successful CMR without incident and subsequently returned home.

The scan was reported 24 h later by the cardiology fellow who suspected a pneumothorax and after confirming their suspicion with the on-call Radiologist immediately phoned the patient to take a further history. This revealed that the patient had in fact felt slightly short of breath for the preceding few weeks associated with an uncomfortable sensation in his back on coughing and inspiration. The patient was asked to return to the emergency department for further clinical assessment where a chest X ray revealed a 3 cm apical pneumothorax. Despite the striking compressional effects of the pneumothorax on the patient’s right ventricle (RV), the patient was clinically well with normal observations and a clinical decision was made to manage the pneumothorax conservatively with close follow up. After 3 weeks and two interval chest X rays, the pneumothorax was found to have resolved.

## Discussion

**Fig. 1:**
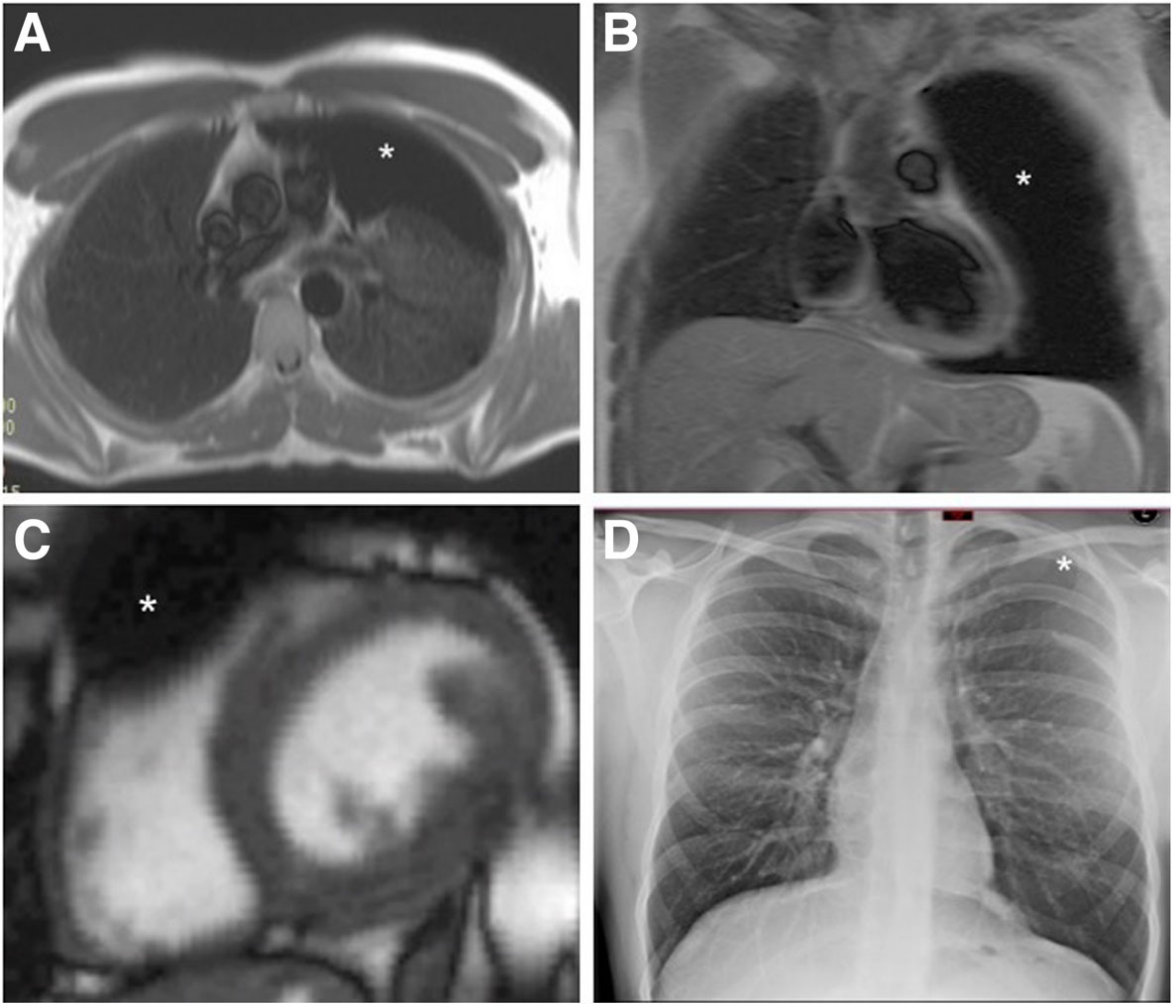
**a** Axial ‘black blood’ CMR slice at the level of the main pulmonary artery showing a large black space devoid of lung markings typical of a large pneumothorax of the left lung. **b** Anterior coronal ‘Black blood’ slice showing complete absence of lung parenchyma in the left anterior chest giving the impression of a much larger pneumothorax, note the absence of mediastinal shift. **c** Short axis stack showing compression of basal right ventricular free wall in diastole. **d** Plain PA chest X Ray showing 3 cm apical pneumothorax. The 2D appearance is much less dramatic than that of panel b


This case highlights several important messages. Firstly, whilst pneumothoraces are only rarely diagnosed on CMR, they are an important, potentially life threatening condition that should not be overlooked. Anyone reporting CMR scans should therefore be aware of the key imaging features (as demonstrated in Fig. 1). Moreover, there is an argument that recognition should be made by supervising doctors at the time of scanning allowing suspected cases to undergo appropriate additional imaging and immediate clinical assessment. Furthermore, this case demonstrates that when a pneumothorax is detected by CMR this modality is actually an excellent technique with which to assess its haemodynamic compression upon the right ventricle in diastole (Additional files 1, 2 and 3).

**Additional file 1:** RVOT cine illustrating striking diastolic and mild systolic compression. (MOV 170 kb)



**Additional file 2:** Short axis cine demonstrating compression of the basal RV in diastole. (MOV 211 kb)



**Additional file 3:**3 chamber cine showing diastolic and to a lesser extent systolic compression of the RV free wall. (MOV 209 kb)


Finally, this case illustrates the importance of paying close attention to extra-cardiac structures whilst reporting CMR scans, with close radiology support for reporting cardiologists [1]. Although examination of extra-cardiac structures is widely adopted by most imaging cardiologists it is not a current CMR training requirement [2] with most imaging centres providing a co-reporting service consisting of a cardiologist and radiologist. This acts to ensure no major extra-cardiac abnormalities are overlooked. Major non-cardiac findings are however only uncommonly discovered on CMR. *Chan* et al. [3] studied 1534 clinical CMR scans and reported the prevalence of non-cardiac pathology as being under 10% with major findings reported as 3% and major new findings less than 0.5%. *Dewey* et al. [4] studied 108 patients undergoing clinical CMR scans and similarly found non-cardiac findings prevalent in 7% with only 2% significant non-cardiac findings [4]. *McKenna* et al. [1] reported a higher prevalence of non-cardiac pathology among a group of 107 volunteers for CMR research, demonstrating potentially significant pathology in 17%. Strikingly not one of the patients in these studies (*n* = 1749) was found to have a pneumothorax. Indeed, we believe these are the first published images of a pneumothorax identified on CMR and certainly the first to illustrate compromised RV filling.

## Conclusions

1. Whilst magnetic resonance imaging is not guideline recommended nor commonly employed for the diagnosis of pneumothorax, those interpreting the examination should be aware of the key imaging features of this potentially fatal condition.
2. Increasing the number of supervised scans is critical to allow rapid identification, assessment and management of unexpected serious pathology.
3. In an era when cardiologists predominantly report CMRs it is important that a co-reporting service with radiologists is available to ensure important non-cardiac findings are not overlooked.
4. If a pneumothorax is incidentally identified, CMR is actually an excellent technique to assess the haemodynamic effects on the right ventricle.
